# Clustering Analysis of Cognitive Profiles of Clinical Groups Using the CAS: An Examination of Japanese Clinical Populations

**DOI:** 10.3390/jintelligence13060071

**Published:** 2025-06-19

**Authors:** Shinji Okazaki, Shiho Okuhata, Masumi Aoki, Hisao Maekawa

**Affiliations:** 1 Institute of Human Sciences, University of Tsukuba, Ibaraki 305-8577, Japan; em_maekawa@human.tsukuba.ac.jp; 2 Faculty of Education, Bukkyo University, Kyoto 603-8301, Japan; s-okuhata@bukkyo-u.ac.jp; 3 Diversity and Inclusion Promotion Office, Tokai University, Hiratsuka 259-1292, Kanagawa, Japan; maoki@tokai.ac.jp

**Keywords:** Cognitive Assessment System, PASS scale, k-means clustering, cognitive profile, ADHD, ASD, comorbid ADHD–ASD

## Abstract

This study examined the distribution characteristics of the standard scores on the Japanese version of the Cognitive Assessment System (CAS)’s Planning, Attention, Simultaneous Processing, and Successive Processing (PASS) scale by clustering the scores using the k-means method, focusing on clinical groups. In Study 1, 140 clinical cases evaluated using the CAS at University A’s educational counseling service were analyzed. The k-means clustering method was applied based on the full-scale standard scores, PASS scale scores, score discrepancies, and subtest scaled scores. Study 2 applied the same clustering method to a clinical group of 91 cases with ADHD, ASD, or comorbid ADHD–ASD, excluding those with intellectual developmental disorders or other disorders. In Study 1, a group with lower full-scale standard scores indicating general intellectual development was identified. Study 2 identified a cluster of cases with ADHD, ASD, or comorbid ADHD–ASD that showed distinct discrepancies among the four standard scores. In addition, there were no significant differences in the diagnoses across clusters. The Japanese version of the CAS provides valid cognitive profile insights in clinical settings, which can aid in planning support interventions beyond clinical diagnosis.

## 1. Introduction

Clarifying the cluster structure in the cognitive testing of children has significant implications for understanding cognitive development and tailoring educational interventions. Combined with the PASS theory of intelligence, this approach offers a more nuanced and comprehensive view of cognitive abilities. This study aimed to examine the distribution characteristics of the standard scores on the Japanese version of the Cognitive Assessment System (CAS)’s (Naglieri and Das 1997) PASS scale using the k-means clustering method, focusing on clinical groups within Japanese populations.

The PASS theory, initially described by Das et al. (1994), identifies four interrelated cognitive processes: planning, attention–arousal, simultaneous processing, and successive processing (Georgiou et al. 2020; Das and Kirby 2022). Rooted in neuropsychological and information-processing research, this framework offers a comprehensive and dynamic alternative to traditional intelligence theories that focus on a single general intelligence factor (g) (Miller et al. 2022; Naglieri and Otero 2019). Over the past two decades, numerous studies have demonstrated the advantages of linking each cognitive process to specific brain areas (Georgiou et al. 2020; Das and Kirby 2022) and providing a robust scientific foundation (Das and Kirby 2022; Okuhata et al. 2009). By emphasizing multidimensional and interconnected cognitive processes, the PASS theory aligns with contemporary perspectives on brain function and plasticity (Cordero-Arroyo et al. 2021; Georgiou et al. 2020).

The PASS theory, along with its associated assessments, such as the Cognitive Assessment System (CAS), provides a nuanced understanding of cognitive functioning. These tools are useful for diagnosing learning disabilities, ADHD, and other cognitive disorders, while also aiding in the differential diagnosis of various developmental conditions (Contena and Taddei 2017; Dunn et al. 2018; Georgiou et al. 2020; Van Luit et al. 2005). Additionally, the PASS theory emphasizes cultural fairness by reducing the biases inherent in traditional IQ tests, such as language dependency and cultural assumptions, thereby ensuring more equitable assessments across diverse populations (Naglieri and Otero 2024). Furthermore, the PASS theory offers a framework for understanding cognitive development across one’s lifespan, making it particularly useful in educational contexts (Miller et al. 2022).

Despite sharing the same diagnostic labels, neurodevelopmental disorders have been empirically shown to encompass multiple subgroups. Cluster analysis offers several advantages in elucidating the structure of intelligence and cognition. It reveals the natural groupings of cognitive abilities, elucidating psychological functions and cognitive deficits (Rivard et al. 2023). This facilitates the data-driven exploration of cognitive profiles and uncovers patterns that might remain hidden in individual analyses. Furthermore, it can identify subgroups with distinct cognitive profiles within heterogeneous populations, such as those with autism and schizophrenia (Ford et al. 2018) and ADHD (Thaler et al. 2013). This analysis enables the detection of overlapping cognitive abilities and boundaries between clusters (Sharma et al. 2019).

In cognitive testing, cluster analysis allows researchers to identify subgroups of children with distinct cognitive profiles. This method highlights the diversity of cognitive abilities among children, which is essential for understanding differences in learning and development. Consequently, educators and psychologists can develop targeted interventions and teaching strategies to address specific cognitive strengths and weaknesses (Ford et al. 2018; Sharma et al. 2019).

According to the PASS theory, neurodevelopmental disorders, despite sharing the same diagnostic label, have been shown to comprise distinct subgroups, as indicated by the profiles derived from cognitive assessments (Keat et al. 2020). Moreover, the presence of comorbid conditions further complicates the cognitive profiles, highlighting the intricate and multifaceted nature of these disorders (Zhang et al. 2022). The combination of cluster analysis and the PASS theory has significant implications for both educational and clinical settings, particularly in terms of aspects of cognition such as attention and planning, which are not directly measured by other cognitive assessments (Naglieri and Pickering 2010). Cluster analysis of psychological functions is a powerful tool for examining complex cognitive processes, such as attention and planning, as it reveals hidden patterns, identifies subgroups with similar profiles, and illuminates the interactions between various psychological functions that may not be easily measured by other methods (Bolin et al. 2014; Taylor et al. 2023). By identifying cognitive profiles through cluster analysis and assessing the PASS processes, educators can tailor instructions to individual students’ cognitive strengths and weaknesses for personalized learning (Goldstein and Naglieri 2008; Mascolo et al. 2014). This approach allows for early identification of cognitive difficulties, enabling timely and/or early interventions that can significantly impact a child’s developmental trajectory.

Therefore, integrating cluster analysis techniques with the PASS theory of intelligence represents a significant advancement in cognitive testing for children. This approach offers a comprehensive, neurologically grounded, and practically applicable framework for understanding cognitive abilities. Moving beyond a single-factor model of intelligence, this approach equips educators and clinicians with tools to more effectively support children’s cognitive development, leading to improved educational and intervention strategies.

In this study, we adopted the widely used k-means algorithm, a data-driven unsupervised learning technique for partitioning a set of data points into k distinct clusters. The objective is to minimize the sum of the squared Euclidean distances between each point and its nearest cluster center. In this context, the centers are selected so that the points within each cluster are as close as possible to the corresponding center (Blömer et al. 2016). This unsupervised method does not rely on distributional assumptions or hypothesis testing, making it suitable for exploratory pattern discovery (e.g., Poletti et al. 2018).

While conventional statistical analyses may suffer from reduced reliability when applied repeatedly to the same dataset, this concern does not extend to k-means clustering. As an unsupervised and exploratory algorithm, k-means is not based on hypothesis testing but on pattern discovery. It is therefore common practice to run it multiple times on the same data to assess the stability and robustness of the identified cluster structure (Jain 2010). Repeated applications—especially with different random initializations—are essential for ensuring the reliability of the clustering results, rather than a source of statistical error.

Despite its simplicity and computational efficiency, several considerations should be taken into account when applying the k-means algorithm (Jain 2010). First, it requires the number of clusters, k, to be predefined, which can be challenging when the underlying data structure is unclear. Second, the algorithm is sensitive to the initial placement of the centroids, which may lead to convergence to suboptimal local minima. Finally, since it updates the cluster centers based on the mean, the algorithm is somewhat susceptible to outliers, which can distort the resulting clusters.

Although the k-means algorithm has known limitations, the standardized nature of the CAS cognitive assessment scores addresses issues related to feature scaling and outlier sensitivity, thereby enabling robust clustering. To determine the optimal number of clusters, we employed the silhouette coefficient (Rousseeuw 1987), which evaluates the validity of clustering by balancing the within-cluster cohesion and the between-cluster separation. Furthermore, to mitigate the influence of the initial centroid selection, we implemented an iterative approach that continues the computation until convergence is achieved.

A Japanese version of the CAS (Naglieri and Das [1997] 2007) has been developed and its reliability and validity have been confirmed (Nakayama et al. 2012). However, to date, no studies have explored cognitive profile patterns using cluster analysis based on the CAS scores in populations with cognitive challenges. To address this gap, the present study applied k-means clustering to analyze the distribution characteristics of the standardized scores on the Japanese version of the CAS’s PASS scale, focusing on clinical groups within Japanese populations.

## 2. Materials and Methods

### 2.1. Participants

A total of 140 clinical cases (111 males, 29 females; age range: 67–216 months) evaluated using the Japanese version of the CAS at University A’s educational counseling service were analyzed. These cases were selected from among children and students who accessed the counseling service at University A between 200X and 200X + 10, consented to participate in this study, and received support based on cognitive profiling using the CAS. The demographic information for all the clinical cases is summarized in Table 1. No significant sex-based differences were observed in the mean age (in months) or mean CAS full-scale scores.

All the participants provided informed consent prior to inclusion in this study. The research was conducted in accordance with the ethical guidelines established by University A and all the procedures were approved by the University’s Research Ethics Committee (Approval No. 2023-178A). Participant confidentiality was strictly maintained, and personal data were anonymized to protect privacy. Furthermore, the participants were informed of their right to withdraw from the study at any time without any repercussions.

### 2.2. Assessment Procedures

The Japanese version of the CAS was administered to all the participants and all the subtests were completed. The assessments were conducted and evaluated by individuals who had undergone appropriate doctoral-level training or were supervised by certified professionals.

### 2.3. Clustering and Statistical Analysis Methods

This study consists of two parts: Study 1, which applies k-means clustering to all 140 consultation cases, without focusing on specific neurodevelopmental disorders, in order to explore the overall cluster structure; and Study 2, which narrows the analysis to cases diagnosed with ADHD, ASD or comorbid ADHD–ASD and applies k-means clustering to examine the cluster structure within these specific groups. The data analyzed in Study 2 overlaps with that in Study 1, specifically the 91 cases of ASD, ADHD, and ASD–ADHD, as shown in Table 1.

In Study 2, the same k-means clustering procedure was applied to a subsample of 91 participants drawn from the original 140 cases analyzed in Study 1. This approach is statistically and methodologically appropriate, as k-means clustering is a data-driven unsupervised learning technique that can be validly applied to both full datasets and well-defined subsamples, provided that the sample size remains adequate and the data structure is preserved (Jain 2010). Since Study 2 focuses on a clinically relevant subgroup (ADHD, ASD, and comorbid ASD–ADHD cases), reapplying the clustering allows for the exploration of specific cognitive profile structures within that population, without violating the assumptions of the method.

As described in the Section 1: Introduction, we applied the k-means clustering algorithm to identify the cognitive profiles in the standardized assessment data. This unsupervised method does not rely on distributional assumptions or hypothesis testing, making it suitable for exploratory pattern discovery (e.g., Poletti et al. 2018). Unlike conventional statistical tests, repeated application of k-means does not increase the type I error risk, as the algorithm is designed to discover latent patterns within the dataset rather than to test predefined hypotheses. The clusters were interpreted as distinct cognitive profiles, with the validity supported through silhouette analysis and repeated runs using random initialization.

#### 2.3.1. Study 1

**Figure 1:**
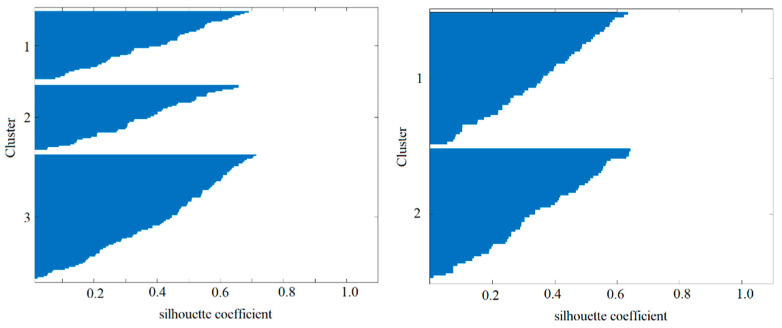
Silhouette plot for Study 1 (**left**) and Study 2 (**right**) after k-means clustering.

The k-means clustering method was applied to all 140 participants using the PASS standard scores, full-scale score (FS), discrepancies between the FS and each PASS standard score (FS-PASS discrepancies), discrepancies among the PASS standard scores (PASS discrepancies), and subtest-scaled scores. K-means clustering was performed with the number of clusters ranging from two to five, and the silhouette coefficient was calculated for each configuration. Consequently, the optimal number of clusters (k = 3) was determined based on the highest silhouette coefficient (0.39). The silhouette plot for Study 1 is shown in Figure 1 (left).

After clustering, a two-way analysis of variance (ANOVA) was conducted with the between-subject factor (three clusters) and within-subject factors (four PASS standard scores and FS) to assess the differences in the cognitive profiles of the identified clusters. Post hoc analysis was conducted using independent *t*-tests to examine the differences in scores between the clusters.

#### 2.3.2. Study 2

Building on the results of Study 1, Study 2 applied the same clustering method to a clinical group of 91 cases diagnosed with ADHD, ASD, or comorbid ADHD–ASD. Cases with intellectual developmental disorders, other neurodevelopmental disorders, or other diagnoses (e.g., cerebral palsy or traumatic brain injury) were excluded. The clustering analysis in Study 2 followed the same procedure as in Study 1, with the number of clusters ranging from two to five. The optimal number of clusters determined using the highest silhouette coefficient (0.35) was two. The silhouette plot for Study 2 is shown in Figure 1 (right).

As in Study 1, ANOVAs were conducted after the clustering to examine the differences in the cognitive characteristics between the two clusters. Additionally, the chi-square test was used to analyze the case distribution across the three disability groups (ADHD, ASD, and comorbid ADHD–ASD).

K-means clustering was performed using MATLAB R2024b (MathWorks Inc., Natick, MA, USA). Subsequent statistical analyses were performed using SPSS version 23 (IBM Inc., Armonk, NY, USA).

## 3. Results

### 3.1. Study 1

**Figure 2:**
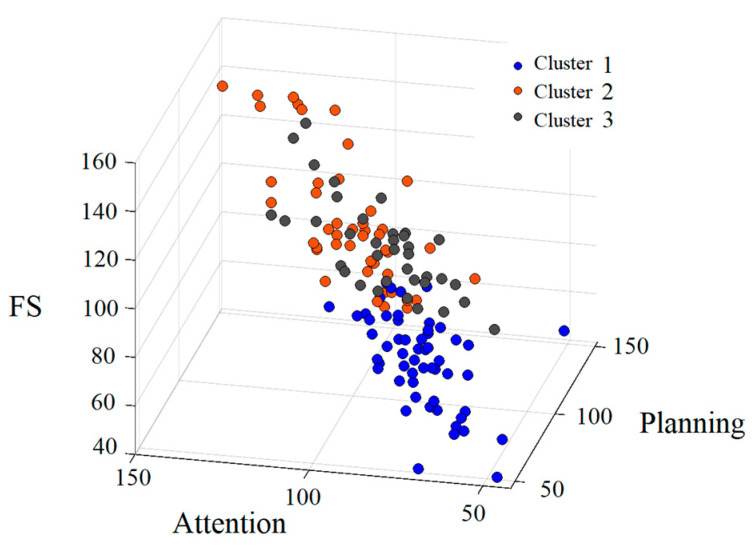
Three-dimensional distribution of the CAS scores: FS, Attention, and Planning across the three clusters.

**Figure 3:**
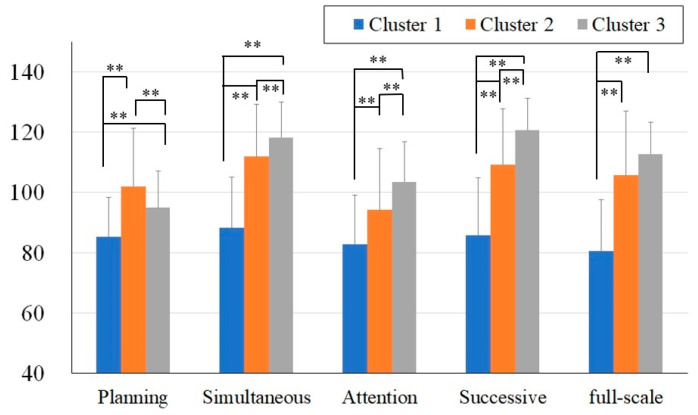
FS and PASS standard scores of the three clusters. Note: **: *p* < .01.

In Study 1, the number of clusters was set to three, based on the highest silhouette coefficient. The results of the chi-square test for the sex distribution were not significant. Additionally, the ANOVA revealed no significant differences in the mean age. The 3D distribution of the standard scores (FS, Attention, and Planning) for the three clusters is shown in Figure 2. The FS and PASS scores for the three clusters are shown in Figure 3. The diagnoses of each cluster are presented in Table 2.

As shown in Figure 2 and Figure 3, Cluster 1 (shown in blue) generally exhibited lower scores across all the measures, whereas the distributions of Cluster 2 (shown in orange) and Cluster 3 (shown in gray) overlapped (Figure 2).

A two-way ANOVA was conducted with the between-subject factors (three clusters) and within-subject factors (PASS standard scores). The analysis revealed a significant main effect of the cluster (F(2, 137) = 101.4, *p* < .01) and the main effect of the PASS standard scores (F(4, 135) = 29.38, *p* < .01). Additionally, the interaction between the clusters and the PASS standard scores was significant (F(8, 125) = 31.44, *p* < .01).

To examine the differences between the clusters, post hoc analyses were conducted using independent *t*-tests. The comparisons between Cluster 1 and Cluster 2 revealed that Cluster 1 had significantly lower across all the PASS standard scores and the FS score (FS: t(101) =−11.57, *p* < .01, Cohen’s d = −2.29; Planning: t(101) = −12.53, *p* < .01, Cohen’s d = −2.49; Simultaneous: t(101) = −7.69, *p* < .01, Cohen’s d = −1.52; Attention: t(101) = −9.99, *p* < .01, Cohen’s d = −1.98; Successive: t(101) = −5.00, *p* < .01, Cohen’s d = −0.99). Similarly, the comparisons between Cluster 1 and Cluster 3 showed significant differences across all the PASS standard scores and FS the score (FS: t(92) = −13.74, *p* < .01, Cohen’s d = −2.90; Planning: t(92) = −6.03, *p* < .01, Cohen’s d = −1.27; Simultaneous: t(92) = −13.49, *p* < .01, Cohen’s d = −2.84; Attention: t(92) = −4.87, *p* < .01, Cohen’s d = −1.03; Successive: t(92) = −12.20, *p* < .01, Cohen’s d = −2.58). In contrast, the comparisons between Clusters 2 and 3 revealed no significant differences in the FS scores. However, significant differences were observed in the PASS standard scores (Planning: t(81) = 5.90, *p* < .01, Cohen’s d = 1.30; Simultaneous: t(81) = −4.44, *p* < .01, Cohen’s d = −0.98; Attention: t(81) = 3.73, *p* < .01, Cohen’s d = 0.83; Successive: t(81) −5.62, *p* < .01, Cohen’s d = −1.24).

Finally, examination of the diagnostic distributions (Table 3) showed that all the cases of intellectual developmental disorder (IDD) and traumatic brain injury (TBI) were exclusively concentrated in Cluster 1. Although no formal statistical analysis was conducted, no discernible pattern of concentration was observed for the other diagnoses within the specific clusters.

To summarize the results of Study 1, clustering based on the Japanese version of the CAS using k-means identified three clusters. Cluster 1 consisted of participants with low scores across all the PASS standard scores and FS. Clusters 2 and 3, while showing the average overall FS, revealed distinct distributions of the PASS scale scores. Individuals with TBI or IDD were predominantly found in Cluster 1, whereas participants diagnosed with ADHD, ASD, or comorbid ADHD–ASD did not show any clear pattern of cluster membership.

### 3.2. Study 2

In Study 2, based on the results of Study 1, 91 cases diagnosed with ADHD, ASD, or comorbid ADHD–ASD were selected according to the exclusion criteria. K-means clustering was performed based on the results of the CAS, and the number of clusters with the highest silhouette coefficient was determined to be two. The results of the chi-square test for the sex distribution were not significant. Additionally, the ANOVA revealed no significant differences in the mean age between the two clusters.

#### 3.2.1. PASS/FS Scores

Study 2 focused on the discrepancies between the FS and each PASS standard score (FS-PASS discrepancies), and the discrepancies between the PASS standard scores (PASS discrepancies). Descriptive statistics for the FS and PASS standard scores for the two clusters are presented in Table 3.

#### 3.2.2. FS-PASS Discrepancies

To compare the discrepancies between the FS and each PASS standard score, a two-way ANOVA with a between-subject factor (two clusters) and a within-subject factor (four FS-PASS discrepancies) was conducted. The results showed that the main effects of the clusters and discrepancies were not significant. However, the interaction was significant (F(3, 264) = 35.53, *p* < .01).

As the interaction effect was significant, a one-way ANOVA was conducted for each cluster to examine the trend of the discrepancies within each cluster. The FS-PASS standard score discrepancies were used as the within-subject factors, followed by Bonferroni post hoc tests. The results revealed significant main effects of the PASS standard scores for both clusters (Cluster 1: F(4, 44) = 61.31, *p* < .01; Cluster 2: F(4, 36) = 7.11, *p* < .01). In the post hoc analysis, significant differences were found in Cluster 1 between the discrepancies in the FS–Planning and FS–Simultaneous (mean difference = 21.78, *p* < .01, 95% CI [15.46, 28.09]), FS–Planning and FS–Successive (mean difference = −20.24, *p* < .01, 95% CI [13.93, 26.56]), FS–Attention and FS–Simultaneous (mean difference = 24.92, *p* < .01, 95% CI [18.6, 31.23]), as well as FS–Attention and FS–Successive (mean difference = 23.39, *p* < .01, 95% CI [17.07, 29.70]).

In Cluster 2, significant differences were observed in FS–Planning and FS–Simultaneous (mean difference = −7.07, *p* < .05, 95% CI [−13.84, −0.31]), FS–Planning and FS–Successive (mean difference = −8.37, *p* < .01, 95% CI [−15.13, −1.60]), as well as FS–Planning and FS–Attention (mean difference = −11.24, *p* < .01, 95% CI [−18.01, −4.48]).

The post hoc analyses using independent *t*-tests revealed that the cluster differences in the FS–PASS discrepancies were all significant (FS–Planning: t(88) = 6.29, *p* < .01, Cohen’s d = 1.33; FS–Simultaneous: t(88) = −5.09, *p* < .01, Cohen’s d = −1.08; FS–Attention: t(88) = 4.52, *p* < .01, Cohen’s d = .96; FS–Successive: t(88) = −6.51, *p* < .01, Cohen’s d = −1.34).

**Figure 4:**
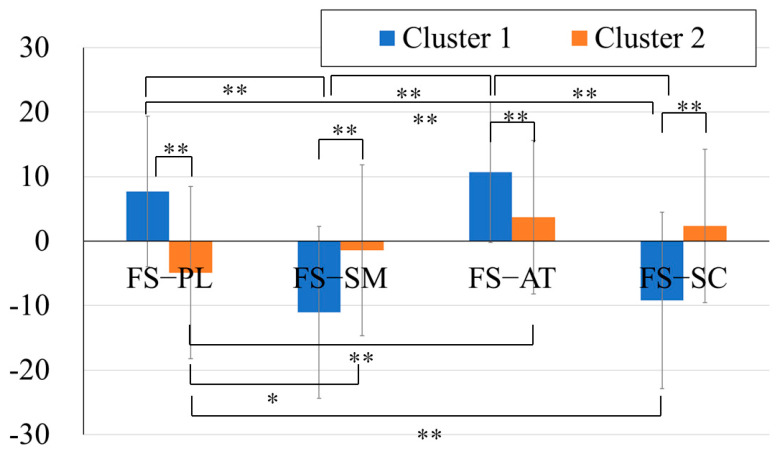
Discrepancies between the FS and each PASS standard score in the two clusters. Note: **: *p* < .01, *: *p* < .05.

In summary, Cluster 1 exhibited higher Simultaneous and Successive scores compared to FS, whereas the Planning and Attention scores were lower. Cluster 2 exhibited smaller FS-PASS discrepancies. These have been highlighted in Figure 4.

#### 3.2.3. PASS Discrepancies

Focusing on the discrepancies between the PASS scales, a two-way ANOVA with a between-subjects factor (two clusters) and within-subjects factors (six PASS discrepancies) was conducted. The results showed a significant main effect of the PASS discrepancies, main effect of the clusters, and interactions (F(5, 445) = 19.63, *p* < .01; F(1, 88) = 107.5, *p* < .01; F(5, 445) = 13.98, *p* < .01, respectively).

A one-way ANOVA was conducted for each cluster to examine the trend of the discrepancies within each cluster. The PASS discrepancies were used as within-subject factors, followed by Bonferroni post hoc tests. The results revealed significant main effects of the PASS standard scores for both clusters (Cluster 1: F(5, 240) = 27.33, *p* < .01; Cluster 2: F(5, 200) = 2.97, *p* < .05). In the post hoc analysis, significant differences were observed in Cluster 1 between the discrepancies in the Planning–Simultaneous and Planning–Attention (mean difference = −24.92, *p* < .01, 95% CI [−35.30, −14.53]), Planning–Simultaneous and Simultaneous–Successive (mean difference = −23.31, *p* < .01, 95% CI [−33.69, −12.92]), Planning–Attention and Planning–Successive (mean difference = 23.39, *p* < .01, 95% CI [13.00, 33.78]), Planning–Attention and Attention–Simultaneous (mean difference = 28.06, *p* < .01, 95% CI [17.67, 38.45]), Planning–Attention and Attention–Successive (mean difference = 26.53, *p* < .01, 95% CI [16.14, 36.92]), Planning–Successive and Simultaneous–Successive (mean difference = −21.78, *p* < .01, 95% CI [−32.16, −11.39]), Attention–Simultaneous and Simultaneous–Successive (mean difference = −26.45, *p* < .01, 95% CI [−36.84, −16.06]), and Simultaneous–Successive and Attention–Successive (mean difference = 24.92, *p* < .01, 95% CI [14.53, 35.31]). In Cluster 2, significant differences were observed only in Planning–Successive and Attention–Simultaneous (mean difference = 12.54, *p* < .05, 95% CI [1.72, 23.35]).

**Figure 5:**
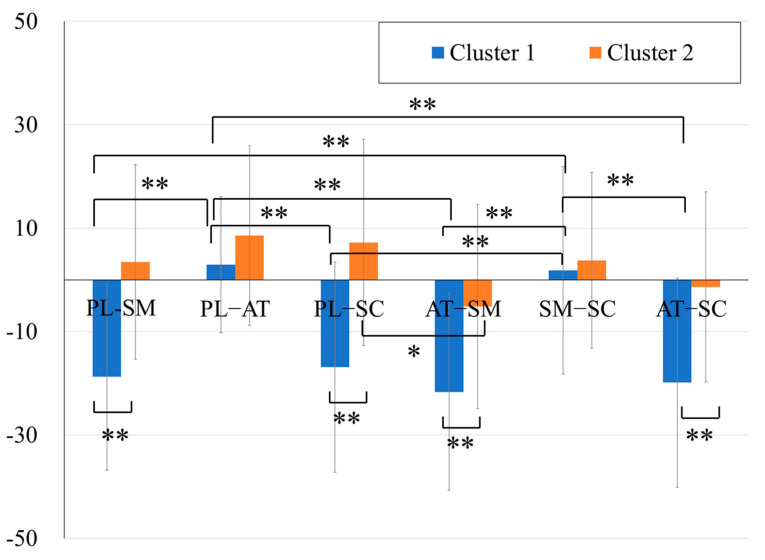
Discrepancies between the PASS standard scores in the two clusters. Note: **: *p* < .01, *: *p* < .05.

The results of the post hoc analysis, conducted using independent *t*-tests to compare the trends of PASS discrepancies between two clusters, revealed the following findings: significant cluster differences were observed in Planning–Simultaneous, Planning–Successive, Attention–Simultaneous, and Attention–Successive (t(88) = −8.57, *p* < .01, Cohen’s d = −1.81; (88) = −8.56, *p* < .01, Cohen’s d = −1.81, t(88) = −6.41, *p* < .01, Cohen’s d = −1.36; t(88) = −7.31, *p* < .01, Cohen’s d = −1.55, respectively) (Figure 5).

**Figure 6:**
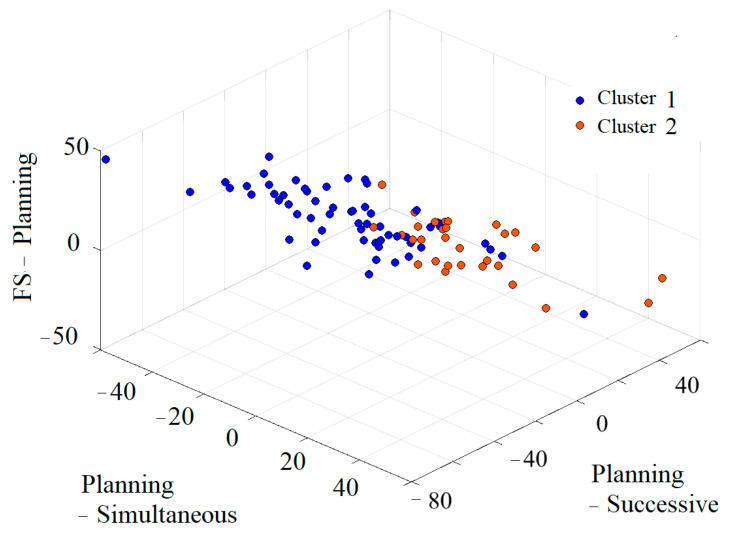

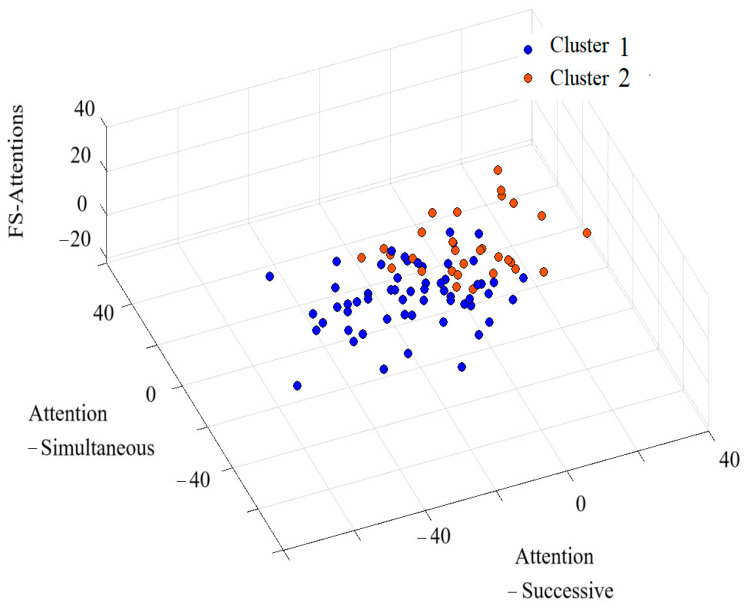
Three-dimensional distribution of the FS-PASS discrepancies and PASS discrepancies for the two clusters. (**Top**): Three-dimensional distribution of the discrepancies for FS–Planning, Planning–Simultaneous, and Planning–Successive. (**Bottom**): Three-dimensional distribution of the discrepancies for FS–Attention, Attention–Simultaneous, and Attention–Successive.

The distributions of the two clusters based on the results are shown in Figure 6. The top panel represents the axes of the FS–Planning, Planning–Simultaneous, and Planning–Successive discrepancies. The bottom panel represents the axes of the FS–Attention, Attention–Simultaneous, and Attention–Successive discrepancies. These results indicate that Cluster 1 can be characterized as a group exhibiting relative weaknesses in Planning and Attention compared to the FS, Simultaneous, and Successive scales. In contrast, Cluster 2 showed less pronounced PASS discrepancies than Cluster 1.

#### 3.2.4. Diagnostic Profiles of the Two Clusters

The chi-square test was conducted to analyze the distribution of diagnoses across the two clusters (Table 4). The results revealed no significant association between cluster membership and diagnosis type. No significant association was observed between sex and cluster membership.

The cluster analysis conducted on a population of individuals with ASD, ADHD, and comorbid ADHD–ASD diagnoses revealed that the population could be divided into two distinct clusters. Cluster 1, characterized by greater discrepancies between the PASS standard scales, exhibited particularly low scores for Attention and Planning. Notably, the cluster classification appeared to be independent of diagnostic labels.

## 4. Discussion

### 4.1. Structure of the Japanese Version of the CAS

The structural validity of the Japanese version of the CAS was supported by the results of Study 1, which employed a k-means clustering analysis of 140 cases. Three distinct clusters that showed clear differences in the distribution patterns of the PASS scale scores and full-scale scores (FS) were identified. Cluster 1 exhibited low scores across all the measures and predominantly included cases of IDD and TBI. Clusters 2 and 3 showed average FS scores but differed in their PASS scale score distributions. These findings suggest that the Japanese version of the CAS is effective in the following. First, identifying severe cognitive impairment associated with IDD and TBI. Second, differentiating cognitive profiles within clinical populations.

The significant differences in the PASS scores between Clusters 2 and 3, despite the similar FS scores, indicate that the Japanese version of the CAS is sensitive enough to capture nuanced cognitive differences within clinical groups (Contena and Taddei 2017; Dunn et al. 2018; Georgiou et al. 2020; Van Luit et al. 2005).

### 4.2. Cluster Characteristics in Specific Clinical Groups of Neurodevelopmental Disorder Populations (ADHD, ASD, and Comorbid ADHD–ASD)

Study 2 focused on 90 cases diagnosed with ADHD, ASD, or comorbid ADHD–ASD. The k-means clustering analysis identified two distinct clusters based on the FS-PASS and PASS discrepancies.

Cluster 1 showed higher Simultaneous and Successive scores than FS and lower Planning and Attention scores. This cluster exhibited significant discrepancies between the PASS scales. Cluster 2 demonstrated smaller overall FS-PASS discrepancies and less pronounced PASS discrepancies than Cluster 1.

Cases without intellectual delay, particularly among those with neurodevelopmental disorders, represent a heterogeneous group even under the same diagnostic label. A cluster study of ADHD using the WISC-IV identified five subgroups based on characteristics such as processing speed and working memory (Thaler et al. 2013). Recent perspectives on ADHD’s heterogeneity have focused on distinguishing between cases with and without irritability, highlighting the role of emotional traits in ADHD (Karalunas et al. 2019).

Although clustering methods were not employed, research within the framework of the PASS theory has shown that ADHD cases with comorbid reading difficulties exhibit significantly lower levels of planning (Zhang et al. 2022). Similarly, different cognitive profiles have been observed within the diagnosis of learning disabilities (Keat et al. 2020). Consistent with these findings, the present study demonstrates that subgroups can also be identified within ADHD (Karalunas et al. 2019; Zhang et al. 2022), ASD, and comorbid ADHD–ASD diagnoses.

Notably, this study found no significant association between cluster membership and the diagnostic categories (ADHD, ASD, and comorbid ADHD–ASD). This finding indicates that the cognitive profiles identified by the Japanese CAS may transcend traditional diagnostic boundaries and provide a more nuanced understanding of cognitive function in various neurodevelopmental disorders.

These results suggest that combining the CAS indices with cluster analysis provides a sensitive and effective approach for identifying meaningful subgroups. This approach offers deeper insights into the cognitive diversity within these populations, paving the way for more targeted and individualized educational and clinical interventions. The key benefits of this approach are as follows:

- Early identification of cognitive difficulties: Aiding proactive measures to address potential challenges.
- Timely interventions: Enabling support that can significantly impact a child’s developmental trajectory.
- Enhanced support for cognitive development: Tailoring strategies to better match individual strengths and weaknesses.
- Improved educational and clinical outcomes: Developing evidence-based strategies for effective teaching and intervention.

### 4.3. The Role of Attention and Planning in Cluster Differentiation

The results revealed consistent differences in the Attention and Planning scores across clusters, as observed in both Study 1 and Study 2. Interestingly, the k-means clustering method used in this study identified two clusters with distinct Attention and Planning profiles, despite their similar overall full-scale scores.

This observation supports the PASS theory’s emphasis on distinct cognitive processes and underscores the importance of examining individual PASS components rather than relying solely on the FS (Naglieri and Otero 2019).

The diagnostic implications of the CAS in children with ADHD highlight the high sensitivity of Planning and Attention to ADHD symptoms, suggesting that these two measures are valuable for assessing specific neurodevelopmental disorders (Qin et al. 2018). However, the highly heterogeneous nature of ADHD, as noted in previous studies (Thaler et al. 2013), complicates its cognitive characterization. In this study, the finding that the cluster characterized by low Attention and Planning scores did not exclusively include ADHD diagnoses further supports this perspective.

A study involving 2146 children aged 5–15 years, all exhibiting learning and behavioral dysfunctions, found that among the four measures of the CAS, Planning and Attention were the most interdependent (Perez-Alvarez et al. 2019). This suggests that difficulties in these two domains are often codependent, a finding further supported by the results of the present study. Furthermore, Planning and Attention are age-independent measures (Perez-Alvarez et al. 2019), enhancing their utility for intervention purposes. Hence, PASS interventions often prioritize training in Planning (strategies) (Das 1994; Das et al. 1994; Mayoral-Rodríguez et al. 2015), as this domain aids the implementation of tailored interventions that can be applied in accordance with the child’s current needs.

Research has indicated that the cognitive profiles in ADHD and ASD are dynamic and evolve in response to development and intervention (Davis and Kollins 2012). Recent studies identified distinct cognitive deficits associated with ADHD in children and adolescents. Childhood ADHD is linked to impairments in vigilance, selective attention, and motor control, whereas adolescent diagnoses are characterized by deficits in cognitive flexibility, working memory, and planning skills (Lau-Zhu et al. 2019; Pascual Zapatero et al. 2024).

In ADHD–ASD comorbidity, unique brain dynamics that differ from those in pure ADHD have been observed, emphasizing the need for tailored diagnostic and treatment approaches (Watanabe and Watanabe 2023). Children with ADHD tend to exhibit lower cognitive abilities than their typically developing peers, particularly in terms of verbal comprehension, processing speed, and working memory (Zhou et al. 2023). Furthermore, children with ADHD and comorbid psychiatric disorders display distinct cognitive profiles compared with those with ADHD alone, with pronounced deficits in the processing speed. These findings highlight the critical need for clinicians to consider developmental differences and comorbidities when designing interventions and assessing cognitive abilities (Genç et al. 2023; Pascual Zapatero et al. 2024).

The heterogeneous nature of the ADHD, ASD, and ADHD–ASD comorbid populations in this study, partly due to the inherent individual cognitive and behavioral differences, may have been influenced by the ongoing interventions. Hence, the diversity in cognitive profiles may have been affected by the intervention outcomes.

These findings underscore the value of applying cluster analysis in conjunction with the PASS theory to identify distinct cognitive profiles. Such an approach could guide the development of targeted interventions and personalized learning strategies (Ford et al. 2018; Sharma et al. 2019). This emphasizes the importance of examining individual cognitive profiles rather than relying solely on diagnostic labels. By doing so, tailored interventions can be developed to address each child’s specific cognitive needs.

### 4.4. General Discussion

The superiority of cluster analysis in applying the Japanese version of the CAS to clinical populations is demonstrated using the following framework:

- Capturing cognitive diversity: Cluster analysis reveals distinct cognitive profiles that do not necessarily align with traditional clinical diagnostic categories such as ADHD or ASD. This approach offers a more nuanced view of individual cognitive functioning, beyond what diagnoses alone can provide.
- Cross-diagnostic perspective: The lack of significant associations between cluster membership and the diagnostic categories suggests that the Japanese version of the CAS, when combined with cluster analysis, offers a perspective on cognitive functioning that extends beyond traditional diagnostic boundaries. This could lead to personalized and effective intervention strategies.
- Identification of specific cognitive patterns: Cluster analysis identified distinct patterns of cognitive strengths and weaknesses, particularly in terms of the discrepancies between the FS-PASS and individual PASS scores. Detailed profiling provides information for targeted interventions and support strategies.
- Potential for refined diagnosis and intervention: The ability to identify subgroups within clinical populations based on cognitive profiles may lead to more precise diagnostic practices and tailored intervention approaches. For example, the individuals in Cluster 1 in Study 2 may benefit from interventions that specifically target Planning and Attention skills.
- Research implications: The clustering approach provides a foundation for future research on the neurobiological basis of these cognitive profiles, potentially leading to a better understanding of the underlying mechanisms of neurodevelopmental disorders.

This framework underscores the value of cluster analysis in advancing both the theoretical understanding and the practical application of the Japanese version of the CAS. This approach holds promise for significantly improving the outcomes in diverse clinical populations by offering a robust tool for bridging research and interventions in clinical settings.

In addition to its practical implications, the methodological strengths and limitations of the clustering approach call for further discussion. We employed k-means clustering for its simplicity, computational efficiency, and suitability for discovering patterns in standardized cognitive assessment data. This technique enabled us to explore the underlying cognitive structures without relying on pre-existing diagnostic frameworks. However, certain limitations must be acknowledged. K-means requires a predefined number of clusters and is sensitive to the initial centroid placement, which can influence the final solution. To address these concerns, we used silhouette analysis to determine the optimal cluster number and performed multiple iterations with random initialization to ensure the robustness. Although k-means does not provide inferential statistics in the traditional sense, it served as an effective exploratory tool for identifying the distinct cognitive profiles in our sample.

## 5. Conclusions

The application of cluster analysis to the Japanese version of the CAS presents a meaningful complement to conventional diagnostic frameworks in clinical contexts. This analytical approach facilitates a more differentiated and nuanced understanding of cognitive functioning, thereby enhancing both clinical practice and empirical research in the field of neurodevelopmental disorders. The integration of cluster analysis with the PASS theory, as exemplified in the present study, constitutes a notable advancement in the assessment of children’s cognitive abilities. It offers a neurologically grounded, theoretically coherent, and practically applicable framework that transcends traditional intelligence models. By moving beyond unitary constructs of intelligence, this methodology equips clinicians and educators with more precise tools to support cognitive development and formulate targeted educational and therapeutic interventions.

The findings of this study underscore the capacity of the CAS to uncover cognitive profiles that extend beyond conventional diagnostic categories, thereby capturing a broader spectrum of cognitive diversity. Importantly, this approach enables the formulation of individualized intervention strategies that respond to specific cognitive patterns, rather than being confined by diagnostic labels alone. Given that the participants in this study were children requiring educational support, the implementation of CAS-based cognitive profiling holds particular promise. Similar findings have been reported in international contexts, suggesting the cross-cultural applicability and relevance of this approach.

Future research should continue to investigate the utility of this integrative framework across diverse populations and cultural settings. Such efforts will contribute to a more refined understanding of cognitive development and facilitate the provision of evidence-based, individualized support for children with varied cognitive profiles.

## Figures and Tables

**Table 1:** Demographic information of participants.

Statistics	Male	Female	
N	111	29	140
Age in months			
Range	67–216	93–194	
Mean (SD)	130 (36.9)	139 (35.5)	n.s.
CAS full-scale score			
Mean (SD)	91.8 (22.4)	88.7 (25.8)	n.s.
Diagnosis			
ADHD	24	2	
ASD	40	11	
ADHD–ASD comorbid	14	0	
IDD	7	6	
SLD	13	2	
TBI	3	3	
CP	1	0	
LBW_HI	0	2	
N/A	9	3	

Note: The abbreviations are as follows: ADHD: attention-deficit/hyperactivity disorder, ASD: autism spectrum disorder, ADHD–ASD comorbid: autism spectrum disorder and attention-deficit/hyperactivity disorder comorbidity, IDD: intellectual developmental disorder, SLD: specific learning disorder, TBI: traumatic brain injury, CP: cerebral palsy, LBW_HI: low birth weight/high risk, n.s.: not significant difference between Male and Female, N/A: No clear diagnosis has been made.

**Table 2:** Diagnostic profiles of the three clusters.

Diagnosis	Cluster 1	Cluster 2	Cluster 3	Total
ADHD	8	9	8	25
ASD	13	16	22	51
ADHD–ASD comorbid	3	8	3	14
IDD	14	0	0	14
SLD	8	6	1	15
TBI	6	0	0	6
CP	0	0	1	1
LBW_HI	1	1	0	2
N/A	4	6	2	12
	57	46	37	140

Note: The abbreviations are as listed in Table 1.

**Table 3:** Descriptive statistics for the FS and the PASS standard scores.

		Planning	Simultaneous	Attention	Successive	FS
Cluster 1	Mean	88.43	110.2	85.29	108.7	97.45
	SD	12.96	16.87	16.19	19.05	17.11
Cluster 2	Mean	107.8	100.7	99.44	96.56	101.4
	SD	19.13	17.29	20.43	18.63	21.32

**Table 4:** Diagnostic profiles of the two clusters.

Diagnose	Cluster 1	Cluster 2	Total
ADHD	13	14	27
ASD	30	20	50
ADHD–ASD comorbid	6	7	13
	49	41	90

Note: The abbreviations are as listed in Table 1.

## Data Availability

The datasets presented in this article are not readily available because the data are part of an ongoing study. Requests to access the datasets should be directed to the corresponding author.

## Abbreviations

The following abbreviations are used in this manuscript:

CAS	Das–Naglieri Cognitive Assessment System
ADHD	Attention-Deficit/Hyperactivity Disorder
ASD	Autism Spectrum Disorder
ADHD–ASD	Autism Spectrum Disorder and Attention-Deficit/Hyperactivity Disorder
PASS	Planning, Attention, Simultaneous Processing, and Successive Processing
IDD	Intellectual Developmental Disorder
TBI	Traumatic Brain Injury
SLD	Specific Learning Disorder
CP	Cerebral Palsy
LBW_HI	Low Birth Weight/High Risk
FS	Full-scale score
